# Molecular Properties and Therapeutic Targeting of the EBV-Encoded Receptor BILF1

**DOI:** 10.3390/cancers13164079

**Published:** 2021-08-13

**Authors:** Julius Maximilian Knerr, Thomas Nitschke Kledal, Mette Marie Rosenkilde

**Affiliations:** 1 Laboratory for Molecular Pharmacology, Department of Biomedical Sciences, University of Copenhagen, 2200 København, Denmark; julius.knerr@sund.ku.dk; 2 Synklino ApS, Rådhusvej 13, 2920 Charlottenlund, Denmark; tnk@synklino.com

**Keywords:** Epstein–Barr virus, cancer, oncogenic virus, G protein–coupled receptor, BILF1, constitutive activity, antiviral treatment

## Abstract

**Simple Summary:**

The Epstein–Barr virus (EBV) is a γ-herpesvirus residing in over 90% of adults worldwide. Besides causing a benign glandular fever (infectious mononucleosis), EBV is also associated with a wide range of different types of cancers. This review will present these malignancies, current therapies, and summarize the present knowledge on an EBV-encoded oncogenic protein called BILF1. As a member of class A G protein–coupled receptors that are intrinsically successful drug targets, BILF1 will be discussed for its potential as future target in EBV-associated diseases. Finally, ongoing development of novel EBV-specific therapeutics is briefly outlined.

**Abstract:**

The γ-herpesvirus Epstein–Barr Virus (EBV) establishes lifelong infections in approximately 90% of adults worldwide. Up to 1,000,000 people yearly are estimated to suffer from health conditions attributed to the infection with this virus, such as nasopharyngeal and gastric carcinomas as well as several forms of B, T and NK cell lymphoma. To date, no EBV-specific therapeutic option has reached the market, greatly reducing the survival prognoses of affected patients. Similar to other herpesviruses, EBV encodes for a G protein–coupled receptor (GPCR), BILF1, affecting a multitude of cellular signaling pathways. BILF1 has been identified to promote immune evasion and tumorigenesis, effectively ensuring a life-long persistence of EBV in, and driving detrimental health conditions to its host. This review summarizes the epidemiology of EBV-associated malignancies, their current standard-of-care, EBV-specific therapeutics in development, GPCRs and their druggability, and most importantly consolidates the findings of over 15 years of research on BILF1 in the context of EBV-specific drug development. Taken together, BILF1 constitutes a promising target for the development of novel EBV-specific therapeutics.

## 1. Introduction

This review on the Epstein–Barr virus (EBV) and its G protein–coupled receptor (GPCR) BILF1 coherently summarizes the knowledge gained within the last years of research. Considering the great impact of EBV on global health with up to 1,000,000 EBV-associated disease cases per year (Table 1), it is of great need to consolidate findings which could drive the development of novel EBV-specific treatments. Additionally, as GPCRs have proven to be highly druggable proteins [1], a significant effort has been put into elucidating the structure, function and druggability of virally encoded GPCRs. First discovered over 15 years ago [2,3], EBV-BILF1 has meanwhile been thoroughly investigated, leading to the revelation of its, among others, oncogenic and immunoevasive properties making it a potential drug target.

## 2. Epstein–Barr Virus

The Epstein–Barr virus (EBV), also known as Human γ-herpesvirus 4, is a ubiquitous virus having infected over 90% of adults globally and forms part of the Lymphocryptovirus genus of the γ-herpesvirinae subfamily [4,5,6]. Discovered in Burkitt’s lymphoma tissue in 1964, it is the first known oncogenic virus [7]. Its morphology is in line with other members of *Herpesviridae*, comprising a monopartite linear ~172 kb dsDNA core encoding for around 100 proteins, a capsid, a tegument and an envelope holding a multitude of glycoproteins [4,8]. Similar to other herpesviruses, EBV has an active lytic and a dormant latent life cycle and thus causes a lifelong infection in the host, in which the virus can switch between latency and lytic reactivation [9]. Upon first contact, the virus enters epithelial cells by endocytosis and in a major histocompatibility complex II (MHC-II) independent (not expressed on epithelial cells) manner. First, the virus attaches to the complement component receptor CD21 with gp350/220 (also via BMRF-2 with integrins) and subsequently fuses with the membrane by the interaction of gH/gL with epithelial integrins αvβ5, αvβ6 or αvβ8 [10] and gB [11]. After successful infection and dissemination from epithelial cells, EBV infects naïve B cells in the lymphoid tissue. These are infected through binding of the EBV glycoprotein gp350/220 to CD21, triggering endocytosis [12]. Then, EBV-gp42 in complex with gH/gL attaches to the MHC-II, which leads to membrane fusion of the host cell and the viral envelope via gH/gL and gB [11].

The lytic infection, in which a host cell is hijacked to produce millions of new viruses, is crucial for the viral dissemination within the host and for transmission to a new host [13]. This cycle is driven by temporally regulated gene expression of immediate-early (IE), early (E) and late (L) lytic genes that encode for lytic proteins [13,14]. IE viral genes are the first genes to be expressed in the cycle and initiate the expression of E viral genes, as well as modulating the host cell environment. In EBV, BZLF1 and BRLF1 take over this role as transcription factors. Early genes are classified as being transcribed before viral replication and are subsequently essential for viral replication, as they, for instance, encode for the viral DNA polymerase (EBV-BALF5) [13]. Moreover, early proteins can also interfere with host cell processes such as major histocompatibility complex I (MHC-I) or MHC-II surface expression, effectively evading the host immune system and, hence, ensuring viral survival. In EBV, the three main early proteins interfering with MHC-I presentation are BNLF2a (inhibitor of transporter associated with antigen processing; TAP), BGLF5 (exonuclease degrading mRNA) and BILF1 (viral G protein–coupled receptor; vGPCR) [15,16,17], the latter of which is the main focus of this review. Proteins interfering with MHC-II surface-presentation are BGLF5, BZLF1 and gp42 [17]. Finally, the late lytic genes, for example, encode for structural proteins important for the assembly of new virions or envelope glycoproteins. They are expressed after the initiation of viral replication and can be inhibited by multiple factors preventing the replication of viral DNA. Examples are gp350/220 (viral entry into host cells), the three capsid proteins (BORF1, BDLF1, BcLF1), as well as MHC-I and MHC-II downregulating protein BDLF3 [18] and viral IL-10 homolog BCRF1 [14].

Besides the lytic cycle, EBV can undergo four different latency programs (0, I, II, III), each defined by a specific restricted expression profile of genes. Compared with the vast number of lytic genes, EBV latent genes are much smaller in number. These latent EBV gene products range from BamHI A region transcripts (BARTs) and microRNAs (miR-BART, miR-BHRF1) over noncoding EBER RNAs (EBER1, EBER2) to nuclear antigens (EBNA1, -2, -3A, -3B, -3C, -LP) and lastly membrane proteins (LMP1, -2A, -2B) and are expressed during different latency programs (I-III) [19,20,21]. In latency 0 (in resting memory B cells), no viral genes are expressed. During cell division, EBV enters latency I, which is defined by sole expression of EBNA1, BART miRNAs and EBERs, to ensure replication and distribution of the viral genome (in the form of an episome) to the daughter cell. Additional expression of LMP1 and LMP2 initiates the latency II program. Expression of the full repertoire of latent genes indicates the latency program III [19]. In the host, persistent latency is generally established in resting memory B cells [19]. Entry to and dissemination from the host (lytic cycle) mainly goes through the mucosal epithelia, consistent with its main route of transmission through oral contact [9].

Predominantly in its latent stage, EBV has been shown to cause a variety of health conditions, which are outlined in the following chapter. While the previously covered definition of latency suggests strict lines between lytic and latent gene expression during latency, the expression of several lytic genes, including BILF1, has been detected in various latent cells (such as nasopharyngeal carcinoma tissue) [22]. This disruption of the traditional view on strict division of lytic and latent gene expression in EBV is extensively covered elsewhere [23].

## 3. EBV Cancers and Standard of Care

Primary infection with EBV, mostly during childhood or adolescence, is known to cause infectious mononucleosis (IM), a generally mild and self-containing glandular fever, in a minority of infected people [5]. More concerning, latent infections with EBV are connected to a large number of cancers in immunodeficient as well as in immunocompetent individuals. As mentioned above, EBV is the first identified oncogenic virus. It is linked to a plethora of health conditions; mostly cancers of B, T or NK cells (Burkitt’s lymphoma (BL), Hodgkin’s lymphoma (HL), post-transplant lymphoproliferative disorders (PTLD) and Mature T- and NK-cell neoplasms (MTNKL)/Peripheral T-cell Lymphoma (PTCL)) [5,24,25]. Some EBV-associated cancers are of epithelial (nasopharyngeal carcinoma (NPC), EBV-associated gastric carcinomas (EBVaGC), lymphoepithelioma-like carcinomas) and, very rarely, of mesenchymal (leiomyosarcoma) origin [5,24]. Moreover, a causative link between EBV and multiple sclerosis (MS) [26,27,28], systemic lupus erythematosus (SLE) [29,30,31] and breast cancer [32,33,34,35] is currently under investigation. Judging by global incidence, EBV-associated gastric carcinomas and nasopharyngeal carcinomas affect most people worldwide with both over 100,000 cases per year (Table 1). Furthermore, if a clear causality between EBV and breast carcinomas can be established, over 500,000 patients yearly could potentially be treated with more efficacious EBV-specific therapeutics (assuming an average of 26% prevalence of EBV in breast carcinomas [32]). Taken together, up to around 1,000,000 people worldwide are diagnosed with diseases with possible EBV involvement every year.

**Figure 1:**
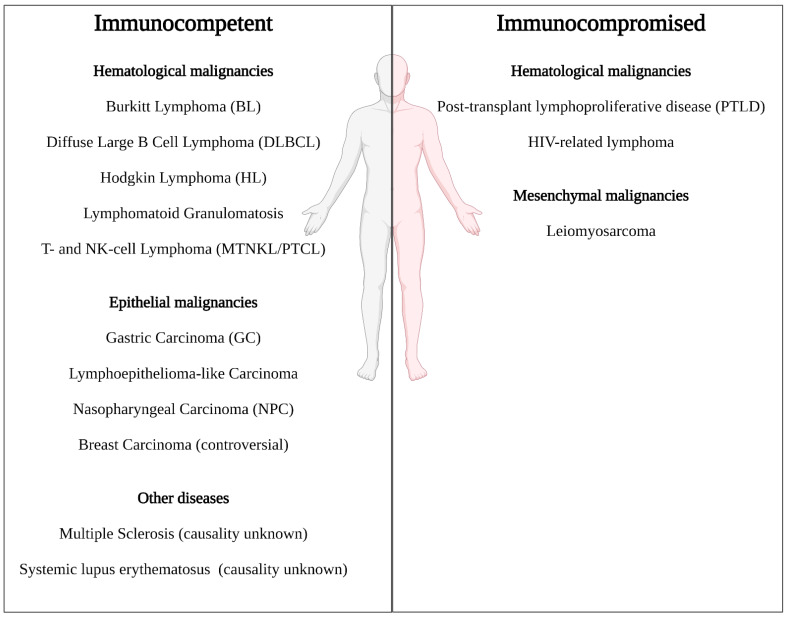
EBV-associated diseases in immunocompetent (**left**) and immunocompromised (**right**) patients. Details are provided in Table 1. Created with BioRender.com.

Figure 1 summarizes EBV-associated malignancies, while Table 1 covers these diseases in detail. It should be noted that the stated rates under ‘Prognosis’ (Table 1) mostly stem from single studies with a limited number of test subjects and not from meta-analyses of multiple studies. Hence, the given values inherently are not representative of every patient and are solely listed to provide a notion on the severity of the respective malignancies. Nonetheless, the OS and PFS found in the literature indicate great room for treatment improvement and immense potential to save lives.

Table 2 complements this summary by providing an overview of the current standard of care (SOC) for these diseases. Currently in surgery, solid organ transplants (SOT), antiviral therapeutics (highly active antiretroviral therapy (HAART), combined antiretroviral therapy (cART), chemotherapy (etoposide, prednisolone, oncovin, cyclophosphamide and hydroxydaunorubicin (EPOCH); cyclophosphamide, hydroxydaunorubicin, oncovin, prednisone (CHOP)) and radiation therapy regimens and a combination of these are the most common approaches. At present, more promising treatments through immunotherapy are gaining traction, i.e., adoptive T cell therapy (ACT), checkpoint inhibiting (CPI) monoclonal antibodies (mAbs) and hematopoietic/autologous stem cell transplants (H/A-SCT)—also in combination with traditional methods. Among many others (cancers, as seen in both tables), one of these newer immunotherapeutical treatments has been examined for multiple sclerosis. Despite lacking a clear causality between EBV and MS, a recent study on EBV-specific T cell therapy in patients with progressive multiple sclerosis resulted in significant clinical improvement and thus could open the door for a wider adoption of alternative therapeutical approaches for MS [105].

## 4. G Protein–Coupled Receptors

### 4.1. Basics

**Figure 2:**
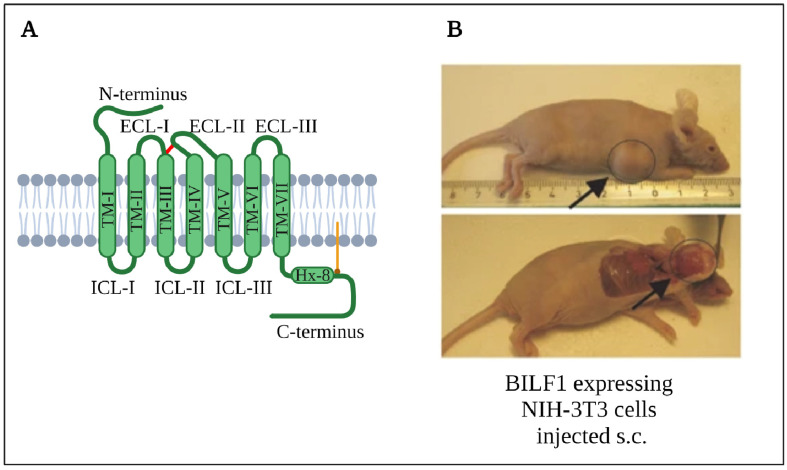
(**A**) Common structure of a class A G protein–coupled receptor (GPCR), such as BILF1. It shows the seven hydrophobic transmembrane (TM) helices, the linking intracellular (ICLs) and extracellular loops (ECLs), and a conserved disulfide bond (red) between TM-3 and ECL-2, Helix 8 and a palmitoylation in orange. (**B**) BILF1 expressing NIH-3T3 cells injected s.c. form tumors in a nude mouse model [122]. Created with BioRender.com.

In the following, the basics on G protein–coupled receptors (GPCRs), also known as seven transmembrane receptors (7TM receptors), are outlined. GPCRs are an essential type of eukaryotic membrane proteins, importantly involved in signal transduction across the cell membrane. According to the International Union of Pharmacology (IUPHAR), GPCRs are divided into five main families: Rhodopsin-like (class A), Secretin (class B), Glutamate (class C), Frizzled/Taste (class F) and Adhesion GPCRs [116]. These receptors consist of an extracellular N-terminus, seven hydrophobic transmembrane helices, joined by three intracellular (ICL) and extracellular loops (ECL), and a cytosolic C-terminus. Class A receptors are the most thoroughly studied receptors and are the focus of this review. They contain a conserved disulfide bridge between transmembrane helix 3 (TM-3) and ECL-2 [117,118], and display a highly conserved DRY-motif essential for signaling at the cytosolic end of TM-3 [118,119]. A schematic depiction of the Class A GPCR structure is presented in Figure 2A. Depending on the specific GPCR, the activating stimuli range from endogenous neurotransmitters, metabolites, hormones or chemokines (chemotactic cytokines) over natural exogenous stimuli such as ions, light and odors to synthetic stimuli such as specific medications [1,117,120,121]. In fact, around 34% of all FDA-approved drugs act on GPCRs [1].

While remaining in an equilibrium of active and inactive conformations in the absence of a ligand (basal or constitutive activity), a GPCR’s active conformation can be stabilized through an agonistic ligand (increased activity to 100% for full agonists and less for partial agonists) [117,120,121]. This allows the GPCR to promote GTP for GDP exchange in the Gα subunit of its cognate heterotrimeric G protein, assembled by a Gα, Gβ and Gγ subunits. Subsequently, the G protein is released from the GPCR and Gα dissociates from the Gβγ dimer, in order to separately initiate amplified intracellular signaling cascades. On the contrary, inverse agonists push the active/inactive conformation equilibrium of the GPCR to the side of inactivation, resulting in a decreased activity compared with the baseline. Additionally, neutral antagonists block the agonist-binding pocket and thus retain basal activity of the receptor and inhibit activation or inactivation through other ligands. Finally, receptors that are active in absence of a ligand are considered constitutively active GPCRs [120,121].

For G proteins, the class of α subunit determines its specificity toward downstream effectors, where Gαi inhibits adenylyl cyclase, Gαs activates adenylate cyclase (triggering the cyclic adenosine monophosphate (cAMP)-dependent pathway), Gαq activates phospholipases C (initiating inositol trisphosphate/diacylglycerol (IP3/DAG) pathway) and Gα_12/13_, among others, interacting with Ras and Rho [123,124,125]. The Gβγ dimer is known to interact with phospholipases, receptor kinases and ion channels [121]. Finally, /GPCRs can recruit arrestins through phosphorylation of their C-terminus by G protein–coupled receptor kinases (GRKs). This inhibits G protein signaling (desensitization), promotes internalization of the GPCRs by clathrin-vesicles and modulates G protein-independent downstream signaling networks [117,121]. Some ligands might initiate a G protein response, while others are more biased toward generating an arrestin response [117].

**Figure 3:**
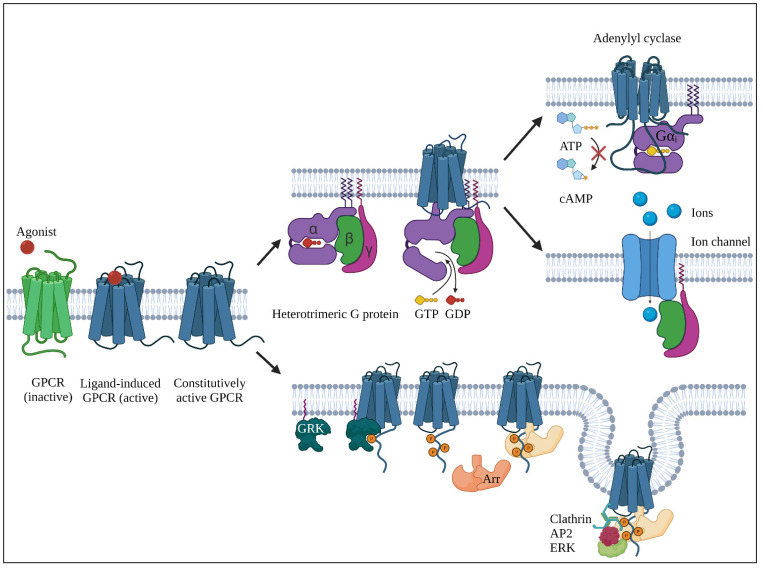
Summary of GPCR activity of constitutively active GPCRs or following binding of an agonist to a receptor. **Top**, the classical G protein pathway. GTP for GDP exchange in the G protein α subunit results in dissociation and modulation of downstream effectors such as Gαi inhibition of adenylyl cyclase and Gβγ activation of ion channels. **Bottom**, arrestin-associated signaling via activated GPCRs. Phosphorylation of the cytosolic C-terminus by a G protein–coupled receptor kinase (GRK) leads to arrestin (Arr) recruitment and activation. This promotes receptor endocytosis via interactions with the clathrin adaptor protein 2 (AP2) complex and activation of extracellular signal-regulated kinase (ERK). Created with BioRender.com. Inspired by Weis et al. [117] and Komolov et al. [126].

The general concepts of GPCR signaling are depicted in Figure 3.

### 4.2. BILF1; a Conserved G Protein–Coupled Receptor in γ1-Herpesviruses

#### 4.2.1. General

BILF1 is a glycosylated viral GPCR (vGPCR) of around 50 kDa (33 kDa unglycosylated) encoded by EBV. This receptor has been shown to mainly associate to the cell membrane of EBV-infected cells [2]. This receptor ticks several boxes of GPCRs, such as signaling through G proteins, containing seven transmembrane helices and displaying conserved cysteine residues in ECLs and the N-terminus. However, BILF1 has an alternative DRY motif at the intracellular end of TM-3. Instead of the well-conserved triad of aspartic acid, arginine and tyrosine (DRY), known among many as rhodopsin-like GPCRs (Class A GPCRs), BILF1 presents an alternative, while similar, DRY-motif (EKT), which maintains the respective charges of the residues [2].

In 2015, 21 orthologs of BILF1 were identified in primate and ungulate γ1-herpesviruses. Among these orthologs, several conserved regions, most intriguingly in the extracellular loops (ECLs) and predominantly in ECL-2, were found [127]. Likewise, these orthologs contain conserved cysteines in ECL-2 and on top of TM-3 (GPCR bridge), and in the N-terminus and the top of TM-7 (chemokine receptor/CKR bridge), both of which are known to form a disulfide bridge in rhodopsin-like 7TM receptors [118,128]. Similarly, NF-κB activation and Gαi signaling are conserved among EBV-BILF1 and studied primate BILF1 orthologs. Moreover, the mentioned study on BILF1 orthologs [127] and a recent study from 2020 [129] also generated data strongly indicating constitutive internalization and recycling of BILF1.

As displayed in numerous studies, DRY/EKT motif in TM-3 is essential for G protein signaling [122,130] and can be disrupted by a K122A substitution [15,122]. In contrast to other vGPCRs such as US28 and ORF74, which primarily signal through Gαq [3,131,132,133] (but also Gαi [131,134,135]), BILF1 solely, ligand independently and constitutively signals via the Gαi class of G proteins, while activating NF-κB and modulating cAMP-response element (CRE) gene regulation pathways [2,3,16,136].

#### 4.2.2. Structure

Unlike other vGPCRs binding CC or CXC chemokines [131] and potentially acting as chemokine scavengers, no endogenous ligand for BILF1 has been found [2,3]. This has led to the general classification of BILF1 as an “orphan” receptor. However, recent studies on elucidating its structure suggest a molecularly evolved ligand-independency of BILF1 [137]. In contrast to common chemokine receptors, this vGPCR displays an unusual conformation. In fact, BILF1 structurally resembles lipid GPCRs rather than chemokine receptors. Here, ECL-2 binds into its own extracellular vestibule, forming a lid. Together with an inward-facing ECL-3, this blocks access of potential ligands to the extracellular binding pocket. In the light of acting as a “self-antagonist”, the previously mentioned conserved regions of ECL-2 among various BILF1 orthologs [127] could prove essential for this lid-forming ability of BILF1. Consequently, the expression “orphan receptor” does not adequately represent the hypothesis that BILF1 “willingly” does not require any ligand for activation. Rather than relying on soluble ligands for activation, it appears that this receptor has acquired several mutations in typical class A GPCR motifs, leading to robust and constitutive activation and signaling [137]. In addition to the previously outlined topologic particularities of BILF1, this study revealed a unique Gαi-binding interface with higher specificity in comparison to endogenous Gαi-binding GPCRs. This could facilitate BILF1′s putative G-protein scavenging properties.

#### 4.2.3. Expression Patterns

BILF1 has been detected in various EBV^+^ cell lines and tissue samples in lytic and latent programs but is mainly considered a lytic protein [3,17,122,138,139,140,141]. Table 3 displays this matter in more detail by listing malignancies with (not ubiquitously) detected BILF1 expression. This receptor has been described as an early lytic protein, though, curiously, BILF1 was shown to progressively interfere with MHC-I antigen presentation throughout the lytic cycle [16,17,138,139].

#### 4.2.4. Cellular Effects

BILF1 has been shown to modulate several systems of the host cell in order to persist in the host. As an initial evasion mechanism, this receptor has been associated to the inhibition of phosphorylation of the RNA-dependent Protein Kinase (PKR) [3]. This interferes with a cellular antiviral defense mechanism, which normally intends to stop protein synthesis and initiate apoptosis of infected cells, hence minimizing virus spread.

As indicated above, BILF1 is able to interfere with host defense mechanisms against EBV through various distinct mechanisms. One major mechanism besides inactivating PKR is the downregulation of MHC-I receptors on the surface of host cells, effectively avoiding a strong CD8^+^ T-lymphocyte (cytotoxic T-cell, CTL) response and, hence, evading quick and efficient eradication of EBV by the host immune system [15,16,136]. In several investigations, it has been indicated that BILF1 must somehow, through its intracellular C-terminal tail, physically interact with the HLA-I molecule (more specifically its heavy chain), as ΔC-terminus mutated versions of BILF1 were not able to downregulate MHC-I surface presentation [16,136]. This downregulation is proposed to occur through BILF1-directed degradation of MHC-I molecules in lysosomes, as lysosomal inhibitors were able to prevent degradation [15]. Even though a first study did not deem signaling ability (intact EKT motif) important for MHC-I downregulation [15], subsequent studies have suggested signaling-independent importance of an intact EKT motif for MHC-I internalization [16,142]. Additionally, besides triggering internalization and degradation of HLA-I molecules, BILF1 also diverts freshly synthesized MHC class I peptide complexes during exocytosis, causing a decrease in membrane-bound MHC-I and proteasome-derived peptide presentation [16]. The mechanism for this effect seems to be independent of the EKT motif and C-terminus.

**Figure 4:**
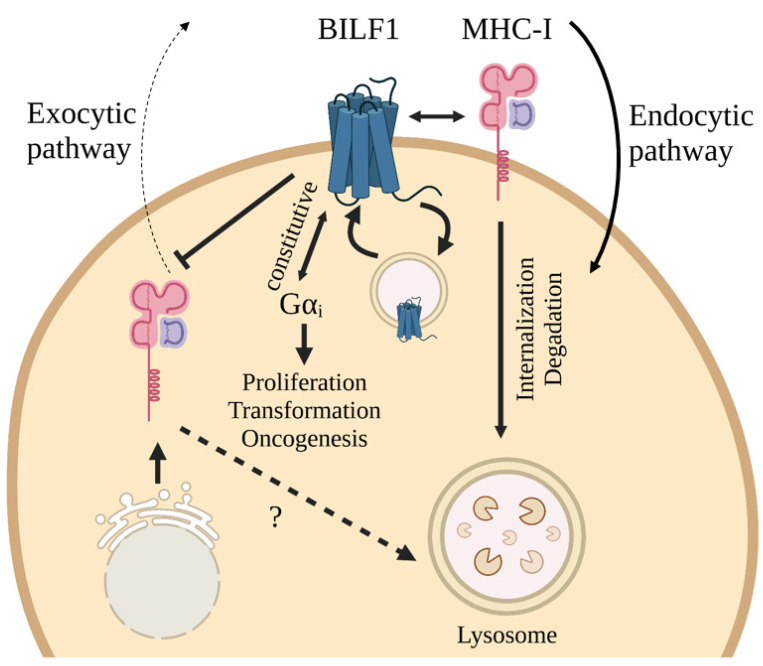
Constitutive signaling and internalization of EBV-BILF1, and its effects on oncogenesis and MHC class I downregulation. In the endocytic pathway, BILF1 physically interacts with MHC-I at the plasma membrane and promotes MHC-I internalization. In the exocytic pathway, BILF1 drives the inhibition of surface expression of newly synthesized MHC-I molecules. Constitutive signaling though Gαi results in cell proliferation, transformation and oncogenesis. Created with BioRender.com.

Furthermore, recent mutational studies on the roles of conserved amino acids (AA) in the extracellular loops (ECLs) revealed that these residues are also directly or indirectly essential for surface downregulation of MHC class I molecules [142]. Interestingly, BILF1 significantly reduces the presentation of HLA-A, HLA-B and HLA-E classes, while having negligible effect on HLA-C subtypes of MHC-I molecules [136]. Through this, EBV could be able to evade strong CTL responses (mainly mediated through HLA-A and -B) while preventing activation of HLA-C-binding NK-cells [136]. Figure 4 summarizes the aforementioned BILF1-associated MHC-I downregulation, while also illustrating constitutive internalization and Gαi signaling through BILF1.

Besides its proposed physical interaction with MHC-I, BILF1 has been described to form heteromers with endogenous chemokine receptors [143,144]. Especially, the interaction between BILF1 and CXCR4 has been surveyed, wherein co-expression of these receptors resulted in impaired CXCL12 binding to CXCR4 [144]. This effect seems to be (BILF1) signaling-dependent and is thought to be achieved through the physical stabilization of an un-inducible CXCR4 conformation within the BILF1:CXCR4 heteromer and/or through BILF1-associated constitutive G protein scavenging, possibly leading to the absence of allosteric modulation of the agonist-binding site. Furthermore, BILF1 assembled into heteromers with the histamine receptor H_4_R, which did not hinder histamine binding, but EKT motif-dependently eliminated H_4_R signaling to CREB. Taken together, it seems more plausible that BILF1 actively inhibits endogenous receptors through its persistent scavenging of Gαi proteins. A BILF1-induced reduction of plasma B cell migration toward organs with high CXCL12 expression has been discussed [144]. This could be advantageous for EBV replication and dissemination and thus could be part of the virus’s sophisticated survival mechanisms [144].

#### 4.2.5. Oncogenesis

In addition to decreasing surface presentation of MHC-I, a study by Lyngaa et al. revealed convincing tumor-promoting effects of BILF1, making BILF1 an oncogene [122]. In this study, Gαi signaling-dependent transformation of NIH-3T3 cells was observed in vitro and in vivo in a mouse xenograft model. In vitro foci formation assays with BILF1^+^ NIH-3T3 cells expressing the wild type (EKT-motif) or one of two mutations (DRY and EAT) showed strong foci induction, which correlated with the amount of constitutive signaling. While the wild type with strong signaling profile induced foci formation with the highest frequency, the DRY mutant (intermediate activity) formed foci with a minimal but significant frequency, and the EAT mutant (no activity) did not produce a significant number of foci compared to the negative control. Another in vitro experiment in this study demonstrated that only the BILF1 wild type, but not the EAT mutant, was able to overcome NIH-3T3 cell contact inhibition, stimulate cell transformation and signaling-dependently increase vascular endothelial growth factor (VEGF) secretion. Correlating to EBV-associated malignancies, increases in VEGF secretion have been linked to both non-Hodgkin’s lymphomas [145] and nasopharyngeal carcinomas [146]. Intriguingly, the bilateral injection of wild type BILF1^+^ cells in nude mice provoked tumor development in 100% of the mice and 87.5% of injection sites, whereas only 60% of the mice and 40% of injections sites resulted in tumor development in mice injected with the EAT mutant of BILF1. The fact that the signaling-deficient EAT mutant was able to induce tumor formation led to the conclusion that the oncogenic properties of BILF1 are not exclusively associated with constitutive signaling through Gαi. Figure 2B displays an exemplary image of a BILF1-induced tumor in this nude mouse model.

Linked to NF-κB activation, BILF1 has recently been found to upregulate the intercellular adhesion molecule-1 (ICAM-1) [139]. As ICAM-1 upregulation has been connected to various types of cancer and might promote cancer metastasis [147], a causative link between BILF1 expression and ICAM-1 upregulation may be valuable to elucidate mechanisms of EBV oncogenicity. Being an NF-κB dependent gene, mutational studies on the NF-κB-binding sites of the ICAM-1 promoter were undertaken, which revealed a significant disruption of the BILF1-linked upregulation of this promoter. Moreover, the cellular level of the endogenous NF-κB inhibitor protein, IκBα, decreased BILF1-dependently, likely resulting in the translocation of NF-κB from the cytoplasm into the nucleus [139].

Summarizing, the picture that BILF1 is painting in terms of association to diseases is becoming quite clear—its role in immune evasion and oncogenesis has been elucidated extensively. The current data suggests that targeting BILF1 with a novel therapeutic might be a way to treat several EBV-associated malignancies. This and the targeting of other GPCRs will be the topic of the next chapter.

### 4.3. Druggability of GPCRs

As GPCRs constitute a large family of receptors imperative for cell signaling, malfunctions can lead to a manifold of different diseases. Consequently, many drugs have been developed to modulate certain GPCRs. Currently, around 34% (481) of all FDA-approved drugs target 107 unique GPCRs [1]. In fact, 69 new drugs have been FDA-approved within the last 5 years, and, as of 2017, 320 drugs, of which 114 are novel drugs acting on 64 novel GPCRs, were under investigation in clinical trials. The diseases and GPCRs in focus are extremely diverse: Metabolic disorders such as hyperparathyroidism (calcium-sensing receptor) [148] and diabetes type 2 (GLP-1 receptor and GPR119 among others) [149]; psychiatric disorders such as schizophrenia (dopamine receptor D_2_) [150]; central nervous system-related diseases such as multiple sclerosis (sphingosine 1-phosphate phosphate receptor 1) [151]; several types of cancer [1,152,153]; and viruses like HIV-1 (CCR5) [154].

Based on the link between previously presented vGPCRs (including BILF1) and tumorigenesis, the general druggability of GPCRs in the context of cancer is briefly outlined [152,153]. Research has revealed several mechanisms through which GPCRs can promote oncogenesis:

- Excess of circulating agonists driving GPCR signaling, which promotes tumor progression (e.g., neuropeptides in small cell lung cancer) [155];
- Mutations in GPCRs or Gα subunit leading to aberrant signaling (e.g., G stimulatory protein (*gsp*), thyroid-stimulating hormone receptor (TSHR) [156]);
- Overexpression of certain GPCRs (e.g., among many others, CXCR4, CCR7 or CXCR1) resulting in increased cancer metastasis, proliferation, cell survival or angiogenesis [157]

The latter mechanism is exploited by the monoclonal antibody ulocuplumab, as it blocks CXCR4 and induces apoptosis in a chronic lymphocytic leukemia (CLL) model [157]. To date, it is not yet FDA-approved. GPCR-targeting anti-cancer drugs with FDA approval are mainly small molecules (sonidegib, vismodegib, cabergoline, raloxifene, brigatinib), but peptides (lanreaotide, degarelix, leuprolide) and mAbs (mogamulizumab, erenumab) are also currently in use [152,153]. The fact that there are already therapeutics targeting GPCRs in cancer—with many more to come—illustrates the general feasibility of GPCR targeting in this context, which could likely also be translated to vGPCR-positive malignancies.

In fact, this is not a new idea: In 1999, Rosenkilde et al. constructed a Zn^2+^ binding double-mutant Kaposi sarcoma herpes virus (KSHV) GPCR ORF74 in order to study the effects of a potential ORF74-specific small molecule drug [158]. Intriguingly, incubation with Zn^2+^ blocked the constitutive signaling of ORF74 with an EC_50_ of 1 µM. This observed inverse agonism indicated the possibility of targeting ORF74 extracellularly with a small molecule ligand, which could potentially interfere with ORF74-driven oncogenesis.

In a different approach, KSHV has also successfully been targeted through immunotoxins binding to lytic glycoproteins and thus killing lytically infected cells in a selective manner [159,160]. Though this can be of benefit in a productive infection, the latent reservoir of this herpesvirus remains untouched, allowing the virus to persist in the host.

Additionally, within the last 20 years, a lot of effort has been put into developing human cytomegalovirus (HCMV) GPCR US28-specific therapeutics, most of which are small molecules [161,162,163,164]. These small molecules displayed inverse agonistic and neutral antagonistic properties, while reaching EC_50_ values in the lower micromolar range. In a recent effort to find additional US28-binding small molecules, over 12 million molecules from the ZINC database were screened in silico, resulting in a library of 98 potential candidates [165]. After conducting inositolphosphate (IP) accumulation and Ca^2+^ mobilization assays, two promising compounds, ZINC36408696 and ZINC38535746, with respective agonistic and inverse agonistic properties, decent potency (0.95 µM and 1.76 µM, respectively) and limited cross-reactivity on other receptors but without CX3CL1 displacing capabilities were identified. In a follow-up study, a new library of commercially available small molecules containing 78 potential US28 modulators was assembled based on the structures of the previously identified compounds [166]. IP accumulation and binding assays revealed several molecules with improvements regarding efficacy and potency compared with the “original” inverse agonist identified in the previous study. Moreover, competitive binding of many of these molecules with CCL2 and CCL4, but not CX3CL1, was observed. These first-in-class studies lay the basis for future development in US28 targeting and modulating small molecules.

Most recently, a research group from the Vrije Universiteit Amsterdam published three highly interesting papers on US28 involvement and targeting in glioblastoma [167,168,169]. Starting chronologically, the first study describes the successful development of a US28-specific single-domain antibody (sdAb), also called nanobody (Nb) or variable heavy fragment (VHH), with sub micromolar affinity [167]. The bivalent version of this nanobody specifically inhibited ligand-dependent and constitutive US28 signaling and hence interfered with US28-driven glioblastoma growth in vitro and in vivo in an orthotopic xenograft mouse model. In a subsequent study [168], the group selected another US28-specific, high-affinity (k_D_ of 2 nM) nanobody and conjugated this with a photosensitizer (IRDye700DX) in order to be used in targeted photodynamic therapy. In vitro binding assays showed improved displacement of CX3CL1 and killing assays on US28-expressing glioblastoma cells generated compelling data in 2D cultures and 3D spheroids. Finally, in the latest study (preprint, not yet peer-reviewed), the previous monovalent nanobody was developed into a bivalent version [169]. This increased the binding affinity to US28 once again (by 10-fold, to 0.2 nM) and retained the ability to inhibit constitutive US28 signaling by 50%, which makes this new construct a partial inverse agonist to US28. Through this inhibition, CMV was partially reactivated in latently infected primary CD14^+^ monocytes and expressed IE genes with only marginal expression of immunoevasins. In the clinic, this could make CMV more detectable to the host immune system, which in turn could promote eradication of the virus.

Similarly, targeting and exploiting a vGPCR with constitutive internalization, a chemokine-ExoA based immunotoxin against HCMV-US28 has been developed [170,171,172]. In these studies, the preferred chemokine to bind US28, CX3CL1, was linked to an ExoA moiety. Additionally, in order to obtain selectivity between US28 and the endogenous CX3CL1 receptor (CX3CR1), a mutated F49A-CX3CL1 was engineered. Due to the constitutive internalizing nature of US28, it has proven to be an ideal target for this kind of treatment, allowing the FTP to efficiently piggyback into the cell. This resulted in a highly potent and selective immunotoxin targeting lytically as well as latently infecting cells, as US28 is expressed in both cycles of CMV. Examples such as the latter clearly indicate the potential of targeting constitutively internalizing viral GPCRs in next-generation antivirals.

The results of all presented studies emphasize the high druggability of the vGPCR US28 and serve as starting points for future development of anti-HCMV medication and as role models for therapeutics targeting of other vGPCRs.

Addressing the need of EBV-specific therapeutics, research on EBV-targeting immunotoxins for nasopharyngeal carcinoma (NPC) has been published [173,174]. These immunotoxins have been shown to selectively kill LMP-1 or LMP-2 expressing NPC cells, respectively, in both in vitro assays and in vivo mouse models. The drawback here is also the incomplete eradication of the virus. As lytic cells do not generally express the latent membrane proteins, these cells would largely remain untouched during the immunotoxin treatment.

BILF1 is the focus of this review and has been suggested as a potential drug target, although research data on BILF1 druggability are scarce. Investigating novel ways to exploit BILF1 in future therapeutics and being aware of the absence of any known endogenous ligands, a research group in 2015 engineered EBV-BILF1 to contain a metal ion binding site through mutations in two transmembrane regions [142], based on the previously mentioned proof-of-concept for ORF74 [158]. This mutated version of BILF1 and the wild type receptor were subsequently incubated with phenanthroline (ZnPhe) or bipyridine (ZnBip) in a Zn^2+^ complex and examined for Gαi signaling activity in an IP3 accumulation assay in HEK-293 cells co-transfected with Gα_qi4my_. The chimeric G protein Gα_qi4my_ couples to Gαi binding GPCRs as a Gαi but signals similar to Gαq, leading to accumulation of IP3 through CRE activation by phospholipase C. While the wild type BILF1 was not affected, ZnBip and ZnPhe decreased the constitutive activity of the double mutant by around 30% with a respective EC_50_ of 2 and 1 µM. Beside the observed inverse agonism, both metal chelators increased the surface expression of the double mutant by 30–40%, while, solely in the case of ZnBip, also promoting surface expression of wild type BILF1 by 15% at 10 µM. Finally, the MHC-I downregulation by wild type BILF1 was inhibited by 10%, and by 15% for the double mutant. In conclusion, this proof-of-concept demonstrated the potential druggability of BILF1 through small molecules, effectively acting as inverse agonists and, hence, possibly inhibiting BILF1′s role in EBV pathology.

## 5. EBV Drug Pipeline

Table 4, Table 5, Table 6 and Table 7 contain a snapshot of the EBV drug pipeline, extracted from GlobalData.com in 2020. As seen in Table 5 and Table 6, most drugs in development are based on cellular immunotherapy or small molecules. Additionally, three respective mAb therapeutics (Table 4) and vaccines (Table 7) are presently being developed.

The antibody pipeline mostly focusses on checkpoint inhibitors against the programmed cell death protein 1 (PD-1) and cytotoxic T-lymphocyte-associated Protein 4 (CTLA-4), but an antibody targeting the thymidine kinase 1 (TK1) is also being researched. In terms of cellular immunotherapy, several different autologous and allogeneic EBV-specific cytotoxic T-lymphocyte treatments targeting several EBV-derived epitopes (EBNA1, LMP1, LMP2, BARF-1) are under development. The majority of the investigated vaccines encode for EBV glycoproteins, which ideally should prevent EBV infections. Interestingly, one vaccine candidate is targeted toward the latent proteins EBNA1 and LMP2, as it is aimed to be used in patients with persistent, recurrent or metastatic nasopharyngeal carcinoma. While the aforementioned therapeutics almost exclusively focus on EBV-derived proteins as targets, the small molecule pipeline aims to interfere with various endogenous proteins, though EBV-specific molecules modulating EBNA1 and BZLF1 are also being investigated.

The fact that several different groups are looking into novel therapeutics to tackle EBV-associated malignancies shows the great need of efficacious and selective strategies and the fact that investing in the EBV-therapeutics market might be a financially attractive opportunity, despite still being in its infancy.

## 6. Conclusions

EBV ubiquitously and persistently infects the grand majority of adults worldwide, and a large number of patients suffer from EBV-associated malignancies every year. Currently, these patients do not have access to EBV-specific therapeutics. Rather, they are treated with common regimens of surgery, chemotherapy, radiotherapy and, potentially, immunotherapy. It is therefore of high interest to discover and develop alternative and EBV-specific treatment strategies. As outlined in this review, there is significant ongoing development of many new and promising drug and vaccine strategies for EBV-associated diseases. Nonetheless, more options are needed. Given the general druggability of GPCRs and the previously successful targeting of another vGPCR (US28), an EBV-encoded GPCR could prove a viable drug target. BILF1 has the potential to be considered a target as such, due to its role in EBV pathology, its internalizing nature and its expression in several EBV-malignancies. Hence, BILF1 should be considered a future drug target for the treatment of EBV-mediated diseases. In order to confirm its potential, more research on BILF1 expression patterns (especially in EBV-malignancies) and druggability must be conducted.

## Figures and Tables

**Table 1:** Description of malignancies in terms of their respective latency program, their EBV-association, the EBV-association depending on the type of the respective disease, the global incidence with EBV-association, the geographical distribution of the disease, the cellular background of the disease, the localization of the disease in the body and the prognosis through the overall survival rate (OS), the progression free survival (PFS), the objective response rate (ORR) and complete remission rate (CRR) where applicable. Inspired by [36].

	EBV-Associated Malignancy	EBV Association	EBV Association—Dependent Upon Type	Incidence with EBV Association	Geography	Cellular Background	Localization	Prognosis
**Immunocompetent patients**	Hodgkin lymphoma (latency II) [37,38,39,40,41]	Developed cnt: 30–50% Developing cnt: 80–90%	Mixed cellularity: 60–80% Nodular sclerosis: 20–40%	29,000/y [41] Up to 56,500 cases and 20,500 deaths in 2018 * [42]	WW	B cells (Reed–Sternberg), (T cells <2%)	Nodal: state 1–3 Extranodal: state 4	CRR: 80–90% [38] 15–20% are resistant/relapse
	Burkitt lymphoma (latency I) [7,37,41,43,44]	Africa: 85%. USA: 15%	Endemic: 95%, sporadic: 25%.	7000/y, 20/100,000 children between 5–9y in sub-Saharan Africa [41]	Endemic: Equa. Africa, New Guinea; Sporadic: WW HIV: WW	B cells	Germinal centers, jaw (young children), breast and abdomen (older children)	Developed cnt: Overall Cure > 90%
								3y OS for chemo-resistant: 7%
	MTNKL/PTCL (latency II) [25,39,45,46,47,48,49,50]	40–50% [49]	AITL: >90%, ANKL: >90%, ENKTCL-NT: 100%, (PTCL-NOS): 30%, SEBV+LOC: 100% [39] 10–30% of all NHL are PTCL/MTNKL [48]	Up to 76,400 cases and 37,500 deaths in 2018 * [48,49,51]	(East) Asia, America, Europe, America	NK cells, T cells	Systemic (AITL, ANKL, SEBV+TLOC); midline nasal/oral cavity, pharynx (ENKTCL-NT),	ENKTCL: 5y OS < 50%. ANKL: Med. OS time 55 days. AITL: Med. 5y OS 32%. PTCL-NOS: Med.5y OS: 20–30% SEBV+LOC: Death within few weeks
	Lymphomatoid granulomatosis (latency I-II) [39,52,53,54,55]	100%		Very rare Prevalence unknown	Western countries	B cells	Lungs, kidneys, skin, CNS	5y OS rate: 40% 50–60% mortality rate
	NPC (latency II) [41,56,57,58,59]	95–100%	Type 1: Squamous (low EBV assoc.) Type 2: Non-keratinizing (high EBV assoc.) Type 3: Undifferentiated (high EBV assoc.)	78,000/y, 80/100,000 mean > 40 years old in Southern China [41] Up to 129,000 cases and 73,000 deaths in 2018 * [56,60]	Asia (Southern China), Africa (north, northwest, central west)	Epithelial cells	Nasal/oral cavity, pharynx	3y OS 86%, 5y OS 79%
	Gastric carcinomas (latency I) [41,61,62,63,64,65]	9–10%	Gastric lymphoepithelioma: 90%, Moderately diff. adenocarcinomas: 7%, Poorly diff. gastric adenocarcinomas: 6%	84,000/y [41] Up to 100,000 cases and 78,000 deaths in 2018 * [65,66]	WW, male predominance	Epithelial cells (Gastric pit cell)	Stomach	Overall general GC: median survival time < 12 months, EBV+ 5y OS: 71%
	Lymphoepithelioma-like carcinomas (latency I ?) [67,68,69,70,71,72,73,74]	Varies	Liver: rare Stomach: >80% Colon: rare Salivary gland ^a^: 90% Lungs ^a^: 64% Thymus ^a^: 44%	Very rare	Mainly Asia	Epithelia	Varies	N/A
	Colorectal carcinomas [75]	Controversial	Controversial	Controversial	WW	Epithelia	Colon	N/A
	Breast carcinoma (latency II) [32,33,34,35]	Controversial [32]	N/A	Potentially Up to 520,000 cases and 162,500 deaths in 2018 * [32,76]	WW, highest EBV Assoc. Asia and America	Mammary epithelia	Breast	5y OS > 90%
	Diffuse large B cell lymphomas NOS (DLBCL) (latency I-III) [50,77,78,79,80,81]	~10% [79]	30–40% of all NHL are DLBCL [78]	Up to 2000 cases in 2018 * [51,78,79]	WW, 10–15% developing cnt. 5% developed cnt.	B cells	Nodal, extranodal (lungs, gastrointestinal tract)	EBV+ DLBCL: 5y OS 25–54%.
**Immunocompromised patients**	Lymphomas (latency I-III) [39,77,82,83,84,85,86,87,88,89,90,91,92,93,94,95,96,97,98,99]	PTLD: 50–80% [92] HIV: 40–50% [39,88]	Hodgkin (HIV): 100% DLBCL (HIV): 30–90%, Burkitt (HIV): 50–60%, PbL (HIV): 80% PbL (PTLD): 30% PTLD in transplant patients: SOT: 1–20%, HSCT: <2% [92]	PTLD: up to 24,640 in 2019 ** [91,92]. HIV: up to 60,000 of newly HIV infected in 2020 will develop EBV+ lymphoma *** [89]; 1–6% of HIV+ patients develop lymphomas each year [90], 50% are EBV+ [88]	WW	B cells (90%), T cells (seldom),	Extranodal, CNS, gastrointestinal	Highly variable (see Table 2) PTLD; 2y OS 83%
	Leiomyosarcomas/smooth muscle tumors (latency I-III) [100,101,102,103,104]	HIV: 85% PT: 98% (B cell > 90%, T cell > 70%) CI: 100% [101]	HIV, PT, CI: Tumor manifestation in <1–5% of each group [101]		WW	Smooth muscle cells	CNS, gut/liver, skin, lungs, larynx, pharynx, adrenal glands, spleen	2y OS 66%, 5y OS 50%

* Calculated from WHO Globocan 2018 report on respective malignancy; ** Calculated from WHO International Report on Organ Donation and Transplantation Activities; *** Calculated from UNAIDS Global HIV & AIDS Statistics 2020; ^a^ low amount of data available; ? not fully elucidated. Abbreviations: ANKL, Aggressive NK-cell leukemia; Assoc, association; AITL, angioimmunoblastic T-cell lymphoma; CI, congenital immunodeficiency; ENKTCL-NT, extranodal NK/T-cell lymphoma (nasal type); GC, gastric carcinomas; Cnt, countries; Diff, differentiated; Equa., equatorial; DLBCL, diffuse large B cell lymphomas; NOS, not otherwise specified; Med, median; MTNKL, mature T- and NK-cell neoplasms; NPC, nasopharyngeal carcinomas; PT, post-transplant; PTCL, peripheral T-cell lymphoma; Pbl, plasmablastic lymphoma; PTLD, posttransplant lymphoproliferative diseases; SEBV+LOC, systemic EBV+ lymphoma of childhood; WW, worldwide.

**Table 2:** Current standard of care (SOC) including prognosis for EBV-associated diseases. For abbreviations, see Table 1.

	EBV-Associated Malignancy	Treatment	Prognosis
**Immunocompetent patients**	Hodgkin lymphoma (latency II) [37,38,39,40,41]	Chemotherapy, radiation therapy, stem cell transplant	CCRR: 80–90% Resistant/relapse: 15–20%
	Burkitt lymphoma (latency I) [7,37,41,43,44,106,107,108,109]	Multiple drug chemotherapy	Overall cure rate in dev. Countries >90%, worse in low-income. 3y OS is 7% for chemoresistant patients
		Combined chemotherapy and immunotherapy (rituximab, α-CD20)	100% overall survival and 95% progression-free survival at 86 months. 3y OS: 89%, 2y OS: 82%
	Mature T- and NK-cell neoplasms/Peripheral T-cell Lymphoma (latency II) [25,39,45,46,47,48,49,50]	ENKTCL-NT: Chemotherapy, radiotherapy	General: 5y OS < 50%. Stage 1 and 2 diseases: 5y PFS 70–72%; 5y OS 61–63%. Stage 1–2: CRR 87%; 5y OS 73%. Stage 3–4: CRR 45%, 5y OS 47% 1y PFS 80%; advances stages: 5y OS 24%, PFS 16%
		ANKL: chemotherapy, HSCT	Median OS: 55 days. 1y OS: 4.4%. Up to median OS 300 days with allo-HSCT and 43% 2y OS (“subacute ANKL”)
		AITL: chemotherapy (CHOP), immunotherapy (CHOP + rituximab/alemtuzumab), high-dose therapy and autologous stem cell rescue (HDT-ASCR)	AITL: Median 5y OS 32% IT(Rituximab)+SCT: ORR 80%, CRR 44%, 2y OS 62% IT(azmab)+SCT: ORR 66–100%, CRR 13–65%, 2y OS < 50% HDT-ASCR: 5y OS 52%
		PTCL-NOS: chemotherapy, HDT-ASCT, no established SOC for relapse/refractory patients	PTCL-NOS: Median 5y OS: 20–30% (< 50% with ASCT) ORR: 50–60%, CRR: 20–30%
		Systemic EBV-positive T-cell lymphoma of childhood: chemotherapy, HSCT	SEBV+TLOC: death within days or weeks of diagnosis
	Lymphomatoid granulomatosis (latency I-II) [39,52,53,54,55]	SOC: corticosteroids, chemotherapy, IFN-α, immunotherapy (rituximab)	5y OS: 40% (SOC), Grade I-II: PFS 5y 56%, Grade III: PFS 4y 40%, CRR 66%, 50–60% mortality rate
		In trials: IFN-α (p with CNS involment), HSCT	80–90% complete remission
	Nasopharyngeal carcinomas (latency II) [41,56,57,58,59]	Surgery, chemotherapy, radiotherapy, 1st line	Phase 2 and 3 trials (*n* = 7) comparing induction chemotherapy and concurrent chemoradiotherapy vs. concurrent chemoradiotherapy: avg 3y (*n* = 5) OS 86% vs. 75% | PFS 76% vs. 64% avg 5y (*n* = 2) OS 79% vs. 73% | PFS 69% vs. 58%
		Immunotherapy (CPI: α-PD1) in recurrent or metastatic disease	Phase 1/2 trials (*n* = 3): avg ORR 27% | avg 1y OS (*n* = 2) 61%, median 16.8 months | avg 1y PFS (*n* = 3) 26%, median 5 months
		CPI α-PD1 with chemotherapy	Phase 1 trial: ORR 91%, 1y PFS 61%
	Gastric carcinomas (latency I) [41,61,62,63,64,65]	General GC: surgical resection with lymphadenectomy, radiotherapy, chemotherapy EBV+ possibly resistant to current chemotherapy options (incl. docetaxel, 5-Fluorouracil)	Overall general GC: OS 20%, median survival time <12 months Recurrence rates (EBV+GC, stages): 0% (I), 21% (II), 33% (III), 83% (IV)
		Immunotherapy (CPI: α-PD1, α-PDL1)	2nd–3rd line of treatment, phase II/III trials over 100 patients (*n* = 2): avg ORR 11.3%, OS time 5.43 months (vs. placebo 0%, 4.14 months) α-PDL1 vs. chemotherapy trial: RR 2.2% vs. 4.3%, OS time 4.6 vs. 5 months
		DNA methylation inhibitors/Demethylating agents	Phase I trial: Significant epigenetic and clinical responses of epigenetic priming with 5-azacytidine (prior to chemotherapy) in patients with locally advanced esophageal/gastric adenocarcinoma
		PI3K inhibitors	Phase III trial: no significant improvement in OS for advanced GC of everlimos in 3rd line treatment Phase I trial: prolonged stable disease with continuous dosing of PX-866
	Lymphoepithelioma-like carcinomas (latency I ?) [67,68,69,70,71,72,73,74]	Surgery, chemotherapy	Varies
	Breast carcinoma (latency II) [32,33,34,35]	Surgery, radiotherapy, chemotherapy, immunotherapy	Generally 5y OS > 90%
	EBV+ diffuse large B cell lymphoma, NOS (latency I-III) [50,77,78,79,80,81]	Antiviral chemotherapy R-CHOP immunotherapy rituximab, durvalumab, nivolumab (α-CD20/PDL1/PD1) EBV CTL, ASCT	5y OS 25–54% >45 y/o: median survival 2 years <45 y/o: CRR > 80%
	Multiple sclerosis [26,27,28,77,105,110,111,112,113,114,115]	Immunotherapy, EBV-specific T-cell immunotherapy in trials	Life expectancy not greatly affected, irreversible disabilities possible, 90% relapsing, remitting MS 10% progressive MS [115]
**Immunocompromised patients**	Lymphomas (latency I-III) [39,77,82,83,84,85,86,87,88,89,90,91,92,93,94,95,96,97,98,99,106,109]	Reduction/cessatation of immune suppression (1st line)	PTLD: ORR 0–73% (biggest study: CRR 37%)
		Chemotherapy	HIV: Burkitt’s lymphoma HAART + chemotherapy: ORR 70%, 3y OS 52% HIV: Hodgkin’s lymphoma cART + chemotherapies: avg 3y OS 51% | 5y OS 76% HIV: Burkitt lymphoma chemotherapy: 4y OS 72% PbL-HIV: median OS 6–19 months PbL-PTLD: median OS 7 months
		Immunotherapy (rituximab) (+ chemotherapy)	PTLD (SOT) chemotherapy + immunotherapy, age <30y (*n* = 55): CRR 69%, 2y OS 83% PTLD: Phase II trials rituximab: ORR 55%, 25% relapse HIV: Hodgkin’s lymphoma ASCT + high dose chemotherapy (relapse): 32-month avg OS 61% HIV: Burkitt’s lymphoma EPOCH-R: 90% OS and 100% PFS at 86 months HIV: Burkitt’s 2y OS: 73%
		Cellular immunotherapy	Phase II trial PTLD (HSCT, SOT) allogeneic EBV-specific CTL by best HLA match: 6-month ORR 52%, 42% CRR
		Transplantation (+ medication)	HIV: Hodgkin’s lymphoma ASCT + high dose chemotherapy (relapse): 32-month avg OS 61%
	Leiomyosarcomas/smooth muscle tumors (latency I-III) [100,101,102,103,104]	Chemotherapy, surgery, antiviral therapy, reduced immunosuppression, adoptive T-cell therapy	PT-SMT: 2y OS 66%, median of death post manifestation 5.5 months PT/HIV-SMT: 5y OS 50%

? not fully elucidated.

**Table 3:** Summary of cell lines and tissue samples with detected BILF1 expression.

Name/EBV-Associated Malignancy	Latency	Type	References
AIDS-related lymphoma (ARL)	I-III	Tissue sample	[140]
Angioimmunoblastic T-cell lymphoma (AITL)	II	Tissue sample	[22,140]
Anaplastic large cell lymphoma (ALCL)	II ?	Tissue sample	[140]
Burkitt lymphoma (BL)	I	Tissue sample	[22,138]
Classical Hodgkin lymphoma, nodular sclerosis (cHL-NS)	II	Tissue sample	[140]
Cutaneous T-cell lymphoma (CTCL)	II ?	Tissue sample	[140]
Diffuse large B-cell lymphoma (DLBCL)	I-III	Tissue sample	[22,140]
Gastric carcinoma (GC)	I	Tissue sample	[22,141]
Mature T- and NK-cell lymphoma (MTNKL)	II	Tissue sample	[22]
Nasopharyngeal carcinoma (NPC)	II	Tissue sample	[22]
Nodular lymphocyte predominant Hodgkin lymphoma (NLPHL)	II	Tissue sample	[140]
Peripheral T-cell lymphoma, not otherwise specified (PTCL-NOS)	II	Tissue sample	[140]
B95-8 (LCL)	III	Cell line	[3,17,140]
HH514.c16 (BL)	I	Cell line	[3]
Jijoye (BL)	I	Cell line	[140]
JY (LCL)	III	Cell line	[3]
KREB2 (LCL)	III	Cell line	[140]
MEC04 (MTNKL)	II	Cell line	[140]
MLEB2 (LCL)	III	Cell line	[140]
Namalwa (BL)	I	Cell line	[3,140]
P3HR1 (BL)	I	Cell line	[140]
Raji (BL)	I	Cell line	[139,140]
SNK6 (MTNKL)	II	Cell line	[140]
Various BL cell lines	I	Cell line	[138]
X50-7 (LCL)	III	Cell line	[3]

? not fully elucidated. Abbreviations: ALR, AIDS-related lymphoma; AITL, angioimmunoblastic T-cell lymphoma; ALCL, anaplastic large cell lymphoma; BL, Burkitt lymphoma; cHL-NS, classical Hodgkin lymphoma, nodular sclerosis; CTCL, cutaneous T-cell lymphoma; DLBCL, diffuse large B-cell lymphoma; GC, gastric carcinoma; LCL, lymphoblastoid cell line; MTNKL, mature T- and NK-cell lymphoma; NPC, nasopharyngeal carcinoma; NLPHL, nodular lymphocyte predominant Hodgkin lymphoma; PTCL-NOS, peripheral T-cell lymphoma, not otherwise specified.

**Table 4:** Antibody pipeline for EBV-linked malignancies.

Indication	Drug Name	Action	Development Stage	Identifier
(Non-) Hodgkin lymphoma	ipilimumab + nivolumab	α-CTLA-4 + α-PD-1	Phase II	NCT01592370
B cell lymphoma	nivolumab	α-PD-1	Phase I	NCT03097939
B cell lymphoma	Viroprev	α-TK1	IND application	http://savoypharmaceuticals.com/viroprev.php, accessed on 12 August 2021

**Table 5:** Cellular immunotherapy pipeline for EBV-linked malignancies.

Indication	Drug Name	Action	Development Stage	Identifier
Nasopharyngeal carcinoma (NPC)		Autologous EBV T Cells	Phase II	NCT00834093
NPC, first-line in combination with gemcitabine + carboplatin	TT10 EBVSTs	Autologous EBV-CTL (EBNA1, BARF-1, LMP)	Phase III	NCT02578641
NPC, relapse/refractory	YT-E001	Autologous EBV-CTL (EBNA1, LMP1, LMP2)	Phase I/II	NCT03648697
NPC	LT-C50	EBV-CTL	Preclinical	https://liontcr.com/pipeline/, accessed on 12 August 2021
Gastric Carcinoma	LT-C60	EBV-CTL	Preclinical	https://liontcr.com/pipeline/, accessed on 12 August 2021
NPC, recurrent/metastatic (platinum-pretreated)	Tabelecleucel + pembrolizumab	Allogeneic EBV-CTL	Phase Ib/II	NCT03769467
CD30+ EBV-lymphomas	TT11x	Autologous EBV-CTL (EBNA1, BARF-1, LMP) + CD30 CAR	Phase I	NCT04288726
EBV+ PTLD	Viralym-M (ALVR105)	Multi-virus specific allogenic T-Cells	Phase II	NCT04693637
Post HSCT opportunistic infections		Autologous or allogenic EBV CTL	Phase II	NCT03159364
EBV+ PTLD after SOT or alloHCT (after failure of rituximab/r+chemo)	tabelecleucel	Allogeneic EBV-CTL	Phase III	NCT03394365
EBV+ PTLD after alloHCT (after failure of rituximab)	tabelecleucel	Allogeneic EBV-CTL	Phase III	NCT03392142
Progressive multiple sclerosis	ATA-188	Allogeneic EBV-CTL	Phase I	NCT03283826
Advanced stage EBV+ malignancies (stage IV gastric carcinoma, NPC, lymphoma after SOC)		Autologous PD-1 knockout EBV-CTL + Fludarabine + Cyclophosphamide + IL-2	Phase II	NCT03044743
Systemic Lupus Erythematosus (SLE)	LUPUS CTL EBV	Autologous EBV-CTL	Phase I/II	NCT02677688
ENKTCL, (PTLD, NPC)	VT-EBV-N	Autologous EBV CTL (LMP1, LMP2a)	Phase II	NCT03671850
ENKTCL, 2nd line	EBViNT	Autologous EBV CTL (LMP2a)	Phase I/II	NCT03789617
Reactivation/infection prevention post cord blood transplant		Autologous EBV CTL	Phase I/II	NCT03594981 NCT01923766

**Table 6:** Small molecule pipeline for EBV-linked malignancies.

Indication	Drug Name	Action	Development Stage	Identifier
NPC, 3rd line, locally recurrent or metastatic	apatinib mesylate	Vascular endothelial growth factor receptor 2 (VEGFR-2) inhibitor	Phase II	NCT03130270
EBV+ Lymphoma	Nanatinostat (VRx-3996)	Histone deacetylase (HDAC) inhibitor	Phase I/II	NCT03397706
Viral cancers/EBV diseases		Inhibition of replication	Lead selection	http://www.virostatics.com/research-and-development/, accessed on 12 August 2021
EBV diseases		BZLF1 activator	Discovery	http://www.vironika.com/pipeline, accessed on 12 August 2021
EBV diseases		EBNA1 inhibitor	Pre-IND	http://www.vironika.com/pipeline, accessed on 12 August 2021

**Table 7:** EBV-specific vaccine pipeline.

Indication	Drug Name	Action	Development Stage	Identifier
Persistent, recurrent or metastatic NPC	MVA vaccine	Recombinant modified vaccinia Ankara (MVA) EBNA1/LMP2 vaccine	Phase II	NCT01094405
EBV infection	mRNA-1189	mRNA-based vaccine (gp350, gH/gL/gp42, gH/gL, gB)	Preclinical	https://www.modernatx.com/pipeline, accessed on 12 August 2021
EBV infection	Vaccine	gp350 blocking	Phase I recruitment	NCT04645147
EBV infection	Vaccine	g42, gH/gL blocking	Preclinical	10.1016/j.immuni.2019.03.010, Patent No. EP3054971, accessed on 12 August 2021

## Data Availability

Data available in a publicly accessible repository.
